# Targeted diversity generation by intraterrestrial archaea and archaeal viruses

**DOI:** 10.1038/ncomms7585

**Published:** 2015-03-23

**Authors:** Blair G. Paul, Sarah C. Bagby, Elizabeth Czornyj, Diego Arambula, Sumit Handa, Alexander Sczyrba, Partho Ghosh, Jeff F. Miller, David L. Valentine

**Affiliations:** 1 Marine Science Institute, University of California, Santa Barbara, California 93106, USA; 2 Department of Microbiology, Immunology and Molecular Genetics, University of California, Los Angeles, California 90095, USA; 3 Department of Chemistry and Biochemistry, University of California San Diego, La Jolla, California 92093, USA; 4 Center for Biotechnology and Faculty of Technology, Bielefeld University, 33615 Bielefeld, Germany; 5 DOE Joint Genome Institute, Walnut Creek, California 94598, USA; 6 Molecular Biology Institute, University of California, Los Angeles, California 90095, USA; 7 California NanoSystems Institute, University of California, Los Angeles, California 90095, USA; 8 Department of Earth Science, University of California Santa Barbara, Santa Barbara, California 93106 USA

## Abstract

In the evolutionary arms race between microbes, their parasites, and their neighbours, the capacity for rapid protein diversification is a potent weapon. Diversity-generating retroelements (DGRs) use mutagenic reverse transcription and retrohoming to generate myriad variants of a target gene. Originally discovered in pathogens, these retroelements have been identified in bacteria and their viruses, but never in archaea. Here we report the discovery of intact DGRs in two distinct intraterrestrial archaeal systems: a novel virus that appears to infect archaea in the marine subsurface, and, separately, two uncultivated nanoarchaea from the terrestrial subsurface. The viral DGR system targets putative tail fibre ligand-binding domains, potentially generating >10^18^ protein variants. The two single-cell nanoarchaeal genomes each possess ≥4 distinct DGRs. Against an expected background of low genome-wide mutation rates, these results demonstrate a previously unsuspected potential for rapid, targeted sequence diversification in intraterrestrial archaea and their viruses.

## Abstract (web summary)

Diversity-generating retroelements (DGRs) are genetic elements that introduce sequence variation within target genes in bacteria and their viruses. Here, Paul *et al*. report the discovery of DGRs in an archaeal virus and in two archaea from marine and terrestrial subsurface environments, respectively.

## Graphical abstract

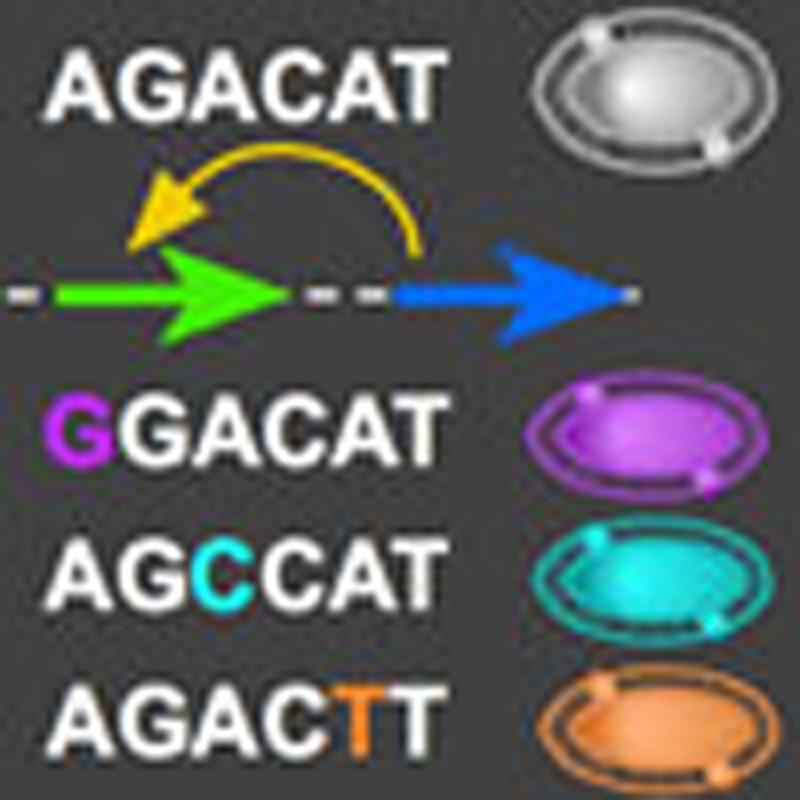

Energy-limited marine and terrestrial subsurface environments harbour a microbial reservoir of exceptional magnitude^1^. Archaea are both numerically dominant^2^ and well adapted to energy limitations faced in various intraterrestrial environments^3^,^4^. Although little is understood about their physiology, metabolism, evolution, or mortality in these environments, current research predicts that they will be characterized by slow growth and low genome-wide mutation rates^5^.

Independent of the sporadic mutation rate, microbial genetic variation can be increased by processes such as gene conversion and horizontal gene transfer. The single most powerful such mechanism known in nature is the diversity-generating retroelement (DGR)^6^,^7^. DGRs use a process called mutagenic retrohoming for the targeted replacement of a variable repeat (VR) coding region with a sequence derived from reverse transcription of a cognate non-coding template repeat (TR) RNA^6^,^7^,^8^,^9^. Crucially, the reverse transcriptase (RT) used is error-prone at template adenine bases^10^, but has high fidelity at other template bases, modulating the rate of diversification to permit rapid exploration of target protein (TP) variants within a recognizable structural framework. Over successive waves of replication, DGR activity leads to rapid evolution of TPs, typically altering ligand-binding specificity^11^ and even permitting phage recognition of novel host ligands^9^. To date, DGRs have been found widely in bacteria and their viruses, but never in an archaeal system.

Because parasitism is expected to be an important driver of evolution and mortality in intraterrestrial archaea^12^, we set out to identify and characterize viruses of anaerobic archaea from one system in the marine subsurface, a methane seep in a California borderlands basin. Our survey uncovers the complete genome of a virus that appears to infect archaea. Remarkably, this genome encodes a complete and apparently active DGR. We examine existing sequence data from archaeal systems, discovering multiple DGRs in the genomes of two subterranean nanoarchaea. These findings demonstrate that subsurface archaea and archaeal viruses maintain a mechanism for generating protein hypervariability within targeted genes, bringing the capacity for massive diversification to the archaea-dominated deep biosphere.

## Results

### A putative archaeal virus encodes a DGR

**Figure 1:**
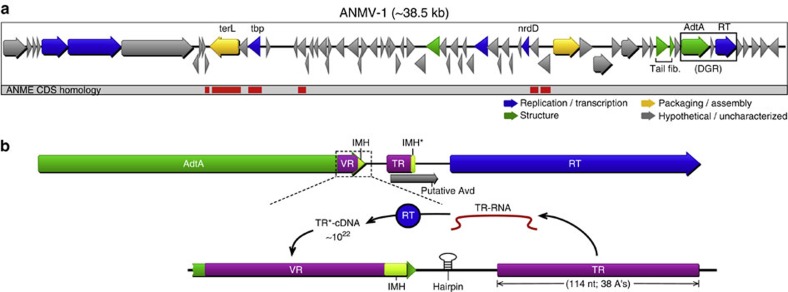
Retroelement-containing ANMV-1 genome obtained from methane seep sediment. (**a**) Annotated coding sequences (CDS) designated by arrows that are coloured according to predicted function. Genes with blast similarity to ANME protein sequences are highlighted in red below each corresponding ANMV-1 locus (Supplementary Table 1). Symbols above selected annotations indicate putative gene names: terL, terminase large subunit; tbp, TATA-box binding protein; nrdD, anaerobic ribonucleoside triphosphate reductase; AdtA, DGR TP; RT, reverse transcriptase. An open box highlights the DGR cassette with flanking putative tail fibres (tail fib.), shown below the genome. (**b**) Putative *cis*- and *trans*-acting features of the ANMV-1 DGR. RT, accessory variability determinant (Avd) and AdtA ORFs are shown as blue, grey and green arrows, respectively. Purple boxes indicate template and variable repeat regions (TR and VR). The IMH and cognate IMH* sites are highlighted in yellow. The expanded DGR view depicts the putative retrohoming target site. Estimated number of nucleotide sequence variants is given above VR (TR* cDNAs), based on theoretical mutagenesis of adenines in TR intermediate RNA.

We collected subsurface sediments from a methane seep at 820 m water depth in Santa Monica Basin. After confirming that these sediments exhibited anaerobic oxidation of methane (Supplementary Fig. 1), we prepared and sequenced a viral metagenome, uncovering a novel and apparently complete viral genome (termed ANMV-1; Fig. 1a). Examination of ANMV-1 coding sequences offered two key lines of evidence that this virus infects an archaeal host. First, the ANMV-1 genome encodes a TATA-box binding protein, an essential component of the transcriptional machinery in archaea and eukarya that is absent from bacteria^13^. Second, the ANMV-1 genome contains six genes that show sequence similarity (*e*-value 10^−7^ to 10^−26^) with proteins from methanotrophic archaea (ANME-1 and ANME-2D) and none with comparable similarity to eukaryotic proteins (Supplementary Table 1). We further hypothesize that ANMV-1’s archaeal host is anaerobic; ribonucleotide reductase activity is essential for phage genome replication^14^, and ANMV-1 encodes an oxygen-sensitive ribonucleotide reductase. In light of the active anaerobic oxidation of methane metabolism observed in the sample from which ANMV-1 was sequenced, the anaerobic archaeal host may belong to an anaerobic methane-oxidizing (ANME) clade.

Analysis of ANMV-1 identified a cassette bearing a RT gene, two 114-bp proximal repeats that vary from each other at positions corresponding to adenines, and a short inverted repeat with potential for hairpin formation (Fig. 1b). Together, these features are hallmarks of a DGR^6^,^7^,^8^,^9^. Since the discovery of these remarkable elements, >300 DGRs have been identified, all within the bacteria and their viruses^15^,^16^. ANMV-1 represents the first identification of a DGR that appears to operate in an archaeal system.

Although the ANMV-1 VR lies within a gene of unknown function (best BLASTp *e*-value >10^−3^, to uncharacterized proteins), the predicted secondary structure of the gene product offered important functional insights. The ANMV-1 DGR target (termed AdtA) shares greatest structural homology (37% of residues modelled with 99% Phyre confidence; r.m.s.d. 1.6 Å; *Z*=13.6) with the major tropism determinant (Mtd) of Bordetella phage BPP-1, a DGR-targeted tail fibre protein responsible for binding host ligands. AdtA contains 21 codons with potential for adenine-specific amino-acid substitutions (versus 12 in Mtd), including nine AAY codons, with potential for >10^18^ variants. Thus, ANMV-1 demonstrates a degree of coding variability that is comparable to bacterial DGR systems^11^ and outpaces the vertebrate immune system’s capacity to generate variants of antibodies or T-cell receptor proteins^17^,^18^. Predicted AdtA structural homology to Mtd is greatest in its C terminus, which corresponds to the C-type lectin (CLec)-fold common to many known bacterial DGR targets^11^,^15^. As in Mtd, the targeted AdtA residues map to partially solvent-exposed sites in the CLec domain (Supplementary Fig. 2). Together, these findings point to a binding-related role for AdtA, and the genomic proximity of the *adtA* gene to phage tail fibre genes (Fig. 1a) suggests host attachment as a possible function.

The discovery of a mechanism for rapid genetic diversification in ANMV-1 raises questions about the distribution and evolution of this virus. We conducted a search for close relatives of the ANMV-1 genome in environmental metagenomic databases, identifying a group of highly similar sequences (Supplementary Fig. 3) found in seafloor sediments of the Nyegga methane seeps, offshore Norway^19^, and in Coal Oil Point hydrocarbon seeps, offshore Santa Barbara, California. Metagenomes from both seeps cover portions of the ANMV-1 DGR cassette, including a closely related and intact RT open reading frame (ORF) from Nyegga seep sediments. These results indicate that ANMV-1 relatives are widespread in methane seeps. Furthermore, the persistence of DGR sequences in related viruses from widely separated ocean basins suggests a selective pressure to maintain the mechanism for targeted protein diversification.

### Two Nanoarchaeota maintain multiple DGRs

**Figure 2:**
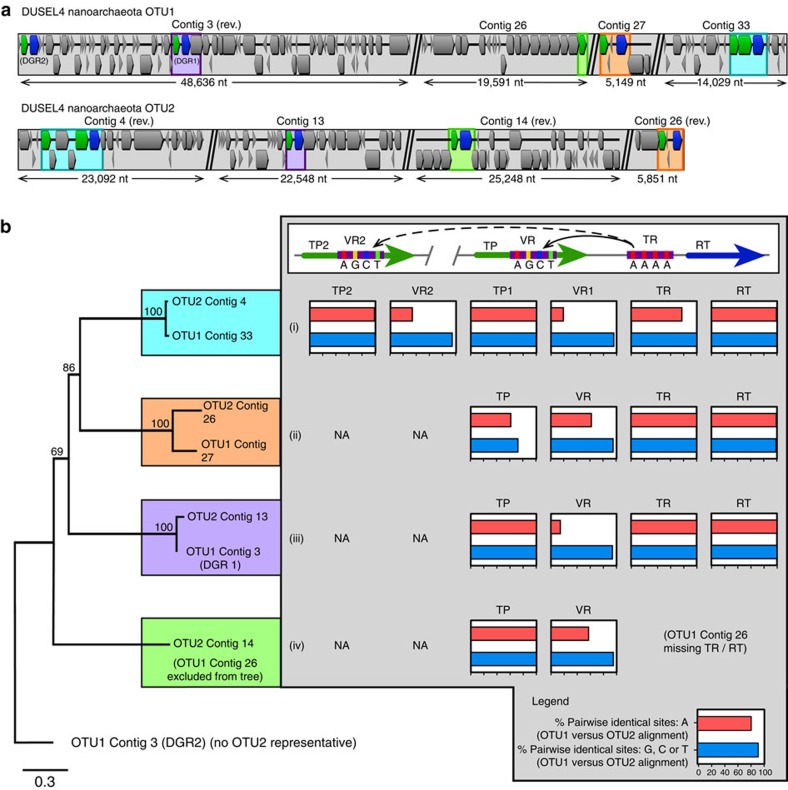
Grouping of DGRs from *Nanoarchaeota*. (**a**) Positions of four DGR cassettes in each OTU, coloured by homology-based groups (note ungrouped OTU1 DGR in grey). Contigs are shown with DGRs on the forward strand (rev., reverse complement). (**b**) DGR groups, ordered by RT and TP homologies. A PhyML tree (left) was constructed with 100 bootstrap replicates (support indicated on branches) from concatenated alignments of TP and RT amino-acid sequences for each complete DGR cassette. Group 4 includes an incomplete DGR for OTU1 contig 26 (missing RT ORF). A schematic for nanoarchaeal DGRs shows the direction of information transfer during targeted mutagenesis. TP and RT genes are shown as green and blue arrows, respectively, while purple boxes indicate variable and template regions (VR and TR). Bar graphs show pairwise similarity between aligned OTU1 and OTU2 sequences for major DGR features, TP, VR, TR and RT. NA (not applicable) indicates that a feature is not found in the DGR.

**Figure 3:**
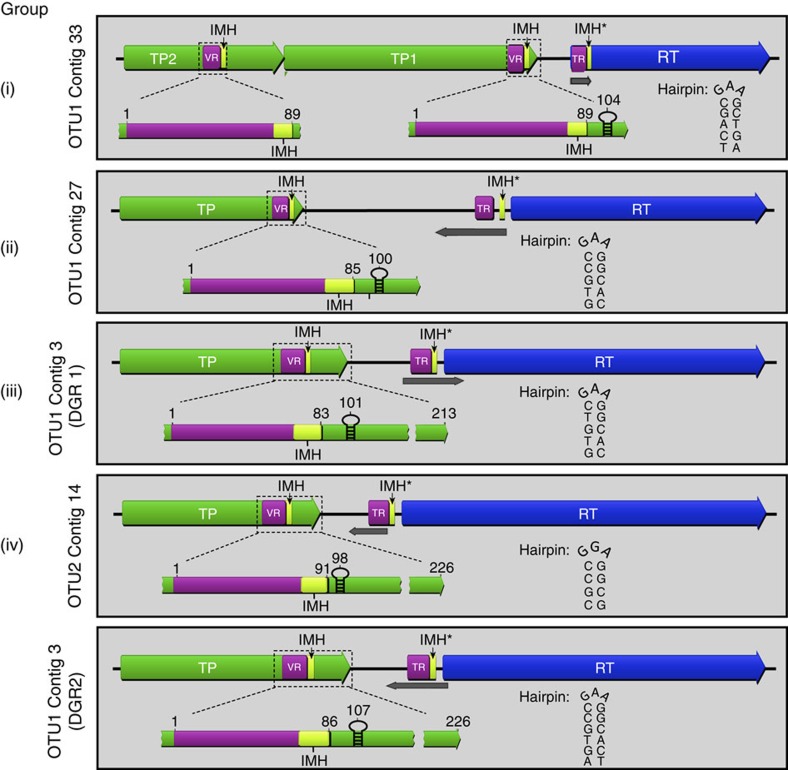
Conserved and putative regulatory features of Nanoarchaeota DGRs. IMH sites (IMH and IMH*) are shown as yellow boxes, and the trinucleotide-loop hairpin is given in an expanded view at right. Dark grey arrows indicate ORFs between RT and TP whose amino-acid sequences have comparable isoelectric point and molecular weight to accessory variability determinant (Avd; pI=9±1; *M*_w_=10±5).

Having identified the first DGR-containing archaeal system, an apparently widespread virus from the marine subsurface, we asked whether distinct DGRs might occur in intraterrestrial archaea themselves. We searched genomic databases for archaeal RT genes and nearby repeats with adenine variability, finding multiple putative DGRs in the two operational taxonomic units (OTU1 and OTU2) of DUSEL4, a clade of uncultivated subterranean *Nanoarchaeota* established from four sequenced cells^20^. Whereas the sequenced genomes of the other known nanoarchaea, *Nanoarchaeum equitans*^21^ (completely sequenced) and *Nanoarchaeote* Nst-1 (ref. 22) (~91% sequenced), so far appear to contain neither DGRs nor RT genes, the DUSEL4 genomes have an abundance, with four distinct (non-redundant) DGR cassettes in a single genome (Fig. 2a). Examination of DUSEL4 RT and TP sequences revealed four distinct groups of DGRs with conserved *cis*- and *trans*-acting features, each with a single representative in both OTU1 and OTU2 (Figs 2b and 3). Intriguingly, a further search within these genomes for VR-containing genes revealed two partial DGRs—consisting only of a target gene, VR, and *cis*-acting elements—in OTU1, the representative with higher estimated genome coverage^20^. Evidence of adenine-directed mutagenesis in these VRs (Supplementary Fig. 4) suggests a history of DGR activity in these sites that do not contain an RT gene, indicating either that the fragments are fossils, left behind when the RT was recruited to a different genomic location or simply lost, or that they are diversified remotely by DGRs elsewhere in the genome.

### Archaeal DGR components have distinct evolutionary histories

**Figure 4:**
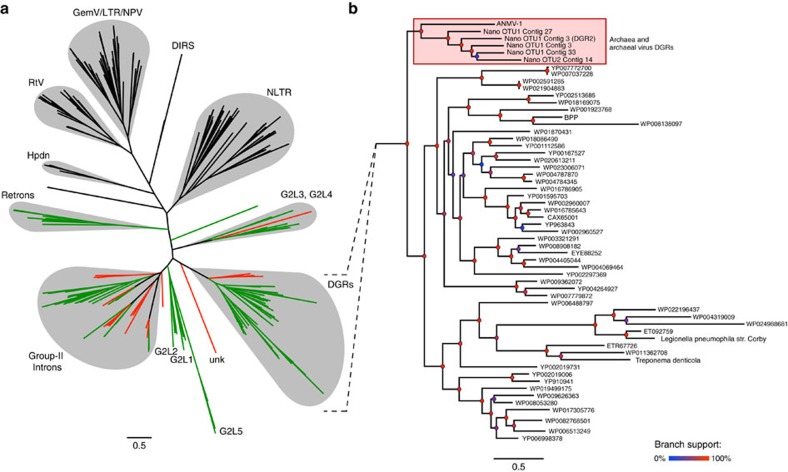
RT phylogeny for archaeal DGRs. (**a**) Maximum-likelihood phylogenetic tree of RT representatives aligned with ANMV-1 and DUSEL4 Nanoarchaeota sequences. Green branches correspond to bacterial and bacteria-derived RTs (from chromosomes, plasmids, mitochondria, chloroplasts and bacteriophage), red branches indicate archaeal and archaeal virus RTs, and black branches represent RTs from eukaryotes and their viruses. Retroelement clades and key representatives are labelled as follows: DGRs, diversity-generating retroelements; DIRS, Dictyostelium retrotransposons; GemV, geminiviridae; G2L, group-II intron-like (G2L are numbered according to Simon and Zimmerly (*24*)); Hpdn, hepadnaviruses; LTR, long terminal repeat retroelements; NPV, nucleopolyhedralviruses; non-LTR, non-long terminal repeat retroelements; RtV, retroviridae; unk, unknown or unclassified. The scale shows substitutions per site. For clarity, bootstrap values are not shown for the full RT tree. (**b**) Expanded subtree view of DGR RT representatives. A red box highlights the archaeal DGR clade. NCBI accession codes are given for representatives in the subtree, but previously described bacterial DGRs are explicitly named. The representative for Bordetella phage BPP is labelled ‘BPP’. Coloured circles at internal nodes indicate branch support.

The possibility that DGRs might not move as a unit led us to examine the evolutionary histories of key DGR cassette components. First, we analysed the phylogeny of the newly identified archaeal DGR RTs. Canonical DGR-type RTs have been shown to form a distinct clade most closely related to bacterial group-II introns^7^,^23^,^24^; while known archaeal RTs are most similar to bacterial group-II and group-II-like introns, they fall outside the DGR clade^24^. We find that the RTs from ANMV-1 and DUSEL4 DGRs lie in a monophyletic group within the DGR clade (Fig. 4a), branching separately from bacterial sequences (97% bootstrap support; Fig. 4b). Underscoring the likelihood that ANMV-1 has an archaeal host, this pattern suggests that ANMV-1 and DUSEL4 DGR RTs share a common archaeal ancestry.

**Figure 5:**
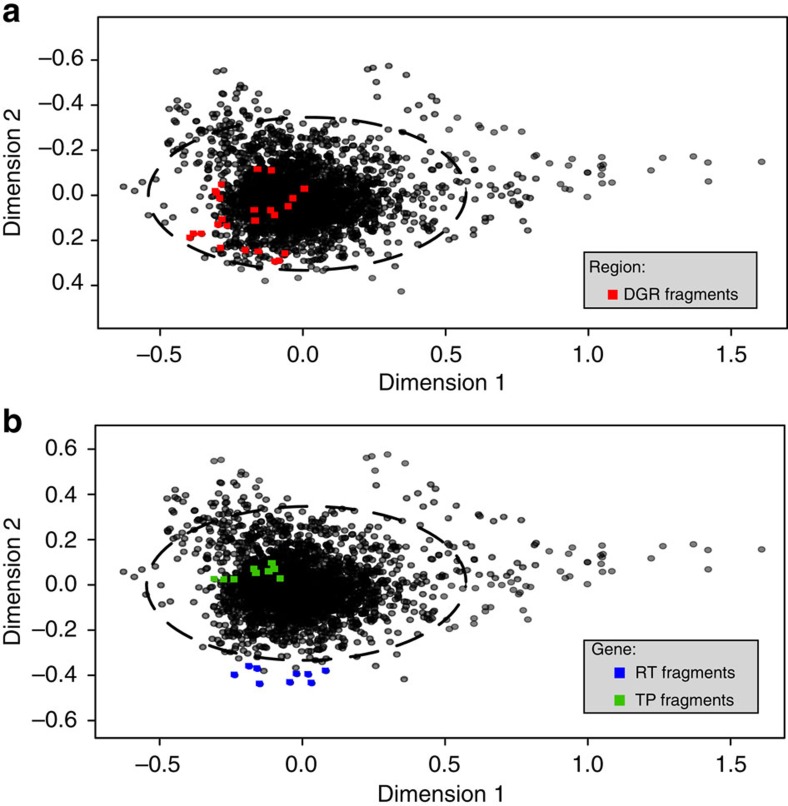
Tetranucleotide distributions of DUSEL4 *Nanoarchaeota*. (**a**,**b**) Non-metric multidimensional scaling plots of tetranucleotide distributions of (**a**) concatenated DUSEL4 DGRs (red) and (**b**) separately concatenated DUSEL4 DGR RT (blue) and TP genes (green), compared with the rest of the DUSEL4 Nanoarchaeota OTU1 and OTU2 genomes (greyscale circles). Each point on the ordination plots represents one 5-kb fragment. Dashed ellipses indicate the 95% confidence region.

We next compared the tetranucleotide composition of DUSEL4 DGRs to that of their host genomes (for individual genome signatures, see Supplementary Fig. 5) at two levels: the concatenated DGRs, and separately concatenated DGR TP genes and RT genes. While TP fragments lie well within the core genomic pattern, RT fragments present as outliers, pulling the overall DGR signature away from the genome core (Fig. 5a,b). Together with the RTs’ phylogenetic relationships, this pattern suggests that DUSEL4 may have acquired its DGR RTs via horizontal transfer, perhaps from another archaeal host. The sequence conservation across multiple DGR RTs in DUSEL4 (Supplementary Fig. 6a) suggests that they have a common source, perhaps a single acquisition followed by repeated gene duplications as new DGRs formed.

### Nanoarchaeal DGRs target orphan genes

Most previously identified bacterial and phage DGRs diversify ligand-binding proteins, predominantly C-type lectin-like^9^,^11^,^15^ or immunoglobulin-like folds^23^,^25^. By contrast, primary sequence analysis of all DUSEL4 *Nanoarchaeota* DGR and DGR fragment TPs reveals that they share no protein sequence homology with either AdtA or any database representatives, but rather constitute a set of orphan genes (Supplementary Fig. 6b); this finding is supported by Phyre analysis, which predicted no structural homology between characterized proteins and any nanoarchaeal TP. Initial structural investigation of one nanoarchaeal TP (OTU1 contig 3 DGR2 TP; Fig. 2b) by circular dichroism (CD) revealed that the purified protein adopts a thermostable fold (*T*_m_~70 °C; Supplementary Fig. 7) even with limited secondary structure (12% α-helix and 25% β-strand)^26^. Pairwise sequence alignments of the nanoarchaeal TPs (Supplementary Fig. 6b) suggest that the targets of groups i–iv are unlikely to share substantial structural homology with each other, raising the possibility that nanoarchaeal DGRs may target a broader range of protein activities than are known for bacterial and phage DGRs.

## Discussion

Comparison of the putative archaeal DGRs with the canonical bacterial and viral DGRs reveals both similarities and distinctive features that may influence DGR function. In Bordetella phage BPP-1, certain *cis*-acting elements appear critical for efficient retrohoming, including (1) an initiation of mutagenic homing (IMH) motif that lies at the 3′ end of VR and an IMH* homologue at the 3′ end of TR; and (2) a short inverted repeat downstream of VR, capable of forming a hairpin/cruciform structure, typically with a GRNA tetraloop^10^. DUSEL4 DGRs appear to maintain versions of these canonical *cis*- acting elements under additional constraints. First, IMH sites in DUSEL4 include a TGGGGT motif, while DUSEL4 IMH* sites carry a corresponding TGGAAT. Second, all DUSEL4 DGR hairpins have highly constrained GRA trinucleotide loops, and each hairpin lies within its DGR’s TP gene, placing this region under selection at the level of both protein structure and DNA sequence. Investigation into the influence of these features on archaeal DGR activity may shed light on differences in the molecular mechanism of DGR retrohoming in bacterial and archaeal systems.

Examination of nanoarchaeal TRs suggests the capacity for individual DGRs to generate 7 × 10^10^ to 9 × 10^12^ variants of their TPs, with no risk of nonsense mutations (Supplementary Fig. 4). Although this range is low by comparison with typical bacterial and viral DGRs, the potential evolutionary impact must be considered in light of the multiplicity of DGRs in DUSEL4 *Nanoarchaeota*; whereas no bacterial or viral genome has been found to harbour >2 distinct DGRs, these nanoarchaea have ≥4. This profusion may enable subterranean nanoarchaea to explore a multidimensional fitness landscape far more rapidly than would sporadic mutation at the low rates observed for other intraterrestrial microbes^5^. Moreover, the fragmentary DGRs elsewhere in OTU1 suggest either that a single nanoarchaeal DGR can concurrently target multiple genes with homologous VRs, or that these DGRs are dynamic, with mobile RT/TR elements recruited from one locus to another over time. In either case, the diversity of nanoarchaeal DGR target sequences so far discovered raises the possibility that these organisms have used DGRs as a general tool for protein engineering—a hint that scientists might be able to do the same.

It is striking that these first discoveries of DGRs in archaeal systems should occur in a virus and in the *Nanoarchaeota*, a phylum associated with parasitism^21^,^22^. Whether the uncultivated organisms represented by the DUSEL4 clade live as obligate parasites remains to be determined; their more important commonality with ANMV-1 may be their occurrence in Earth’s subsurface. While massive and low-risk protein diversification offers clear advantages to any organism caught up in the Red Queen’s race, the occurrence of a DGR in the globally distributed virus ANMV-1 and the proliferation of DGRs in subterranean nanoarchaea suggests that these elements may confer additional selective advantages in a compartmentalized and energy-limited subsurface environment.

## Methods

### Study site and sampling

Paull’s Pingo is a seafloor mound feature (latitude 33.799° N and longitude 118.646° W, depth ~820 m) formed by the expansion of subsurface methane hydrate^27^. We accessed active methane seeps at the pingo to collect sediment cores using deep submergence vehicle *Alvin*, during R/V *Atlantis* Leg AT15-53 (September 2009). Sediment core processing was conducted shipboard in an anaerobic chamber, flushed with a nitrogen headspace. One sediment core was subsectioned between 5 and 15 cm (relative to seafloor) and dedicated to methane-amended incubations. Two subsamples of 60 ml sediment were homogenized with 20 ml of sterile, anoxic artificial seawater medium^28^. Incubations with the homogenized sediments were prepared in 120-ml serum vials, under a 40-ml headspace of ~3% CH_4_ and 97% N_2_. Incubations were amended with ^13^C-labelled methane (99 atom-% ^13^C) as an exogenous tracer to track methane oxidation (Supplementary Fig. 1). Stable isotope ratios (*δ*^13^C) for CO_2_ were measured by isotope ratio mass spectrometry (Thermo Finnigan Delta XP Plus in continuous flow mode). After 1 month of enrichment, the incubation was terminated and viruses were purified for DNA sequencing.

### Virome purification and DNA sequencing

Incubation slurry samples (1:2 sediment:aqueous phase) were used for virus particle purifications. Samples were vigorously homogenized by vortexing (15 min), followed by centrifugation (10 min, 500*g*). Supernatant was filtered (0.22 μm) to separate viruses from cells. Viruses were concentrated and viral DNA was extracted as previously described^29^. Briefly, virus particles were concentrated via caesium chloride density gradient ultracentrifugation (2 h, 22,000 *g*, 4 °C) and treated with DNase-I. DNA was extracted by cetrimonium bromide (CTAB)-chloroform and phenol-chloroform separation. Before viral DNA amplification, a 16S PCR assay to screen for cellular DNA contamination was performed with universal bacterial primers Bact27F (5′- AGAGTTTGATCCTGGCTCAG -3′) and Bact1492R (5′- GGTTACCTTGTTACGACTT -3′). Following this check, we performed Phi29 polymerase multiple displacement amplification (MDA) using the Illustra Genomiphi HY DNA Amplification Kit (GE Healthcare). Thermal cycling steps for denaturing template DNA, polymerase amplification, and post-amplification enzyme inactivation were performed according to the manufacturer’s specifications, except that the MDA amplification reaction was incubated for 2 h instead of 4 h (2 h, 30 °C). Amplified product was pyrosequenced on 454-titanium plates at the Broad Institute, as part of the Moore Marine Phage Metagenome Initiative^30^. Metagenomic reads can be obtained under the NCBI BioSample accession code PRJNA47435.DV-ANM1.

### Read preprocessing, binning, and assembly

Raw sequencing reads were first scanned for sequencing primers, which were identified and removed using TagCleaner^31^. The reads were then preprocessed to remove low-quality sequence following the method of Hurwitz *et al*.^32^, using a custom R script. Preprocessing included, first, removal of any reads with ambiguous (N) bases; second, removal of the shortest 2.5% and longest 2.5% of reads; third, removal of reads with mean quality score >2 s.d. below the mean; and finally, de-replication with CD-Hit 454 (ref. 33).

Reads that passed preprocessing and quality control (QC) steps were subjected to *de novo* assembly using CAMERA’s meta-assembler^34^. As this assembler does not permit user manipulation of read overlap parameters, we compared the meta-assembler output with a custom reassembly approach using Geneious v7.0 (Biomatters Ltd) with the following parameters: minimum overlap 35 bases, overlap pairwise identity 90% and index word length 12 nt. The ANMV-1 contig described in this study was generated from the meta-assembly and aligned globally with 97.7% pairwise nucleotide similarity to a contig obtained by the second custom *de novo* assembly. PCR screening confirmed the authenticity of the ANMV-1 DGR cassette in both template and MDA-amplified viral DNA, using primers that partially overlap TP, RT and VR/TR regions: ANMVdgrF (5′- AGGCGATGCAGACGAATGGC -3′) and ANMVdgrR (5′- TTGCCCAGAGTTACACCGAGCG -3′).

### Metagenome annotations

Prediction of open reading frames was performed using Glimmer3 (ref. 35) with default parameters. Translated ORF sequences were annotated via CAMERA-HMM and BLASTp^36^ searches against the following databases: TIGRfam, Pfam, COG and NCBI-nr (*e*-value <10^−3^). To determine which ORFs from ANMV-1 genome share similarity to viral and prophage sequences, we compared our contig’s translated ORFs with the ACLAME prophage-specific database^37^. To assess similarity to proteins from anaerobic methane-oxidizing archaea, we inspected NCBI-nr BLASTp results for ANME protein hits (uncultured archaeon, ANME-1; ‘*Candidatus* Methanoperedens nitroreducens’, ANME-2D; and uncultured archaeon, Gfoz37D1). A BLASTn survey was conducted against environmental metagenomic databases, including NCBI metagenomic sequences (env_nt), Moore Marine Virus Metagenomes^30^ and Pacific Ocean Virome sequences^38^, to find representatives sharing high nucleotide similarity (*e*-value <10^−20^; 28-nt word size) with ANMV-1.

The putative DGR TP of ANMV-1, AdtA, was analysed using Phyre2 (ref. 40) to find functional representatives based on secondary structural homology. Residues of TP that aligned with high confidence to the CLec fold region of the Mtd protein *Bordetella* phage BPP-1 (Phyre confidence >90%) were used to predict a three-dimensional model. Residue positioning was assessed by Ramachandran analysis and C-terminal variable residues were mapped from the primary sequence onto the predicted structure using Geneious v7.0 (Biomatters Ltd).

### Comparative analysis of Nanoarchaeota genomes

We identified DGR-like RTs via BLASTp searches against the NCBI-WGS database. For an initial proxy of DGR repeat features, we used the EMBOSS tool Dotmatcher^40^ to perform a dotplot analysis of homologous regions with moderate proximity (±5 kb) to RT. TR/VR regions were confirmed from candidates that comprised mostly adenine-specific variability, with at least 10 adenine-specific mismatches, with respect to one strand, and no more than 2 non-adenine mismatches in 100 bp of aligned sequence.

DGR-containing sequences that were analysed in this study are from single-cell genomes belonging to DUSEL4 *Nanoarchaeota*, which were broadly described as part of a genome and metagenome annotation study on ‘microbial dark matter’, published elsewhere^20^. DUSEL4 *Nanoarchaeota* representatives were previously assigned into two OTUs comprising four single-cell genomes. We describe *Nanoarchaeota* DGRs with reference to their occurrence in combined single-cell sequence assemblies: OTU1 (genomes AAA011-G17 and AAA011-L22) and OTU2 (genomes AAA011-J02 and AAA011-K22). To confirm the presence of multiple distinct DGRs in one single-cell genome, we aligned OTU1 sequences with contigs from *Nanoarchaeota* AAA011-G17, which has the highest genome completeness of the DUSEL4 representatives^20^.

Nanoarchaeota RT sequences were aligned using ClustalW^41^ with sequences containing the catalytic RT domain, representing DGRs, group-II introns, retrons, long terminal repeats (LTRs), retroviruses, non-LTR elements and retroplasmids. The alignment was compared with a position-specific scoring matrix for the RVT-1 protein family (PF00078), and was manually realigned to conserve motifs considered essential for RT activity. Trees were constructed in MEGA v5.2 (ref. 42) using PhyML^42^ with the model LG+G+F. In addition, a PhyML tree was constructed from concatenated alignments of RT and TP amino-acid sequences to compare sequence similarities amongst Nanoarchaeota DGR cassettes.

### TP expression and purification

Coding sequences of nanoarchaeal TPs were synthesized with codons optimal for expression in *Escherichia coli* (GENEWIZ, Inc.) and cloned into a modified pET28b expression vector with an N-terminal His-tag followed by a PreScission protease cleavage site. Construct integrity was confirmed by DNA sequencing. TPs were expressed in *Escherichia coli* BL21-Gold (DE3) cells. Bacteria were grown with shaking at 37 °C to an optical density (OD600) of 0.6–0.8 and then cooled to room temperature, followed by induction with 0.5 mM isopropyl β–D-1-thiogalactopyranoside. Bacteria were grown with shaking at room temperature for 5–6 h further, then harvested by centrifugation (25 min, 4,000*g*, 4 °C); the bacterial pellet was frozen at −80 °C.

Cells were thawed and resuspended in buffer A (300 mM NaCl, 50 mM Tris (pH 8) and 5 mM β-mercaptoethanol; 20 ml l^−1^ of bacterial culture) supplemented with 1 mM phenylmethylsulfonyl fluoride (PMSF). The bacteria were lysed by sonication and the lysate was centrifuged (30 min, 35,000 *g*, 4 °C). The following steps were performed at 4 °C. The supernatant was applied to a column containing His-Select Nickel affinity gel (Sigma, 1 ml of resin per 20 ml of bacterial lysate), which had been equilibrated with buffer A. The column was washed with five column volumes of buffer B (300 mM NaCl, 20 mM Tris (pH 8) and 5 mM β-mercaptoethanol) containing 20 mM imidazole, and the TP was eluted with buffer B containing 250 mM imidazole. The His-tag was removed by PreScission protease cleavage (1:50 TP: protease mass ratio) overnight at 4 °C. Cleaved TP was separated from non-cleaved proteins by applying the sample to a His-Select Nickel affinity gel column (Sigma) and collecting the flowthrough. The TP was further purified by gel filtration chromatography (Superdex 75) in 300 mM NaCl, 20 mM Tris (pH 8) and 1 mM dithiothreitol. Purified protein was concentrated to 2 mg ml^−1^ using ultrafiltration (10 kDa MWCO Amicon, Millipore); the concentration of TP was determined using a calculated molar extinction coefficient at 280 nm of 28,880 M^−1^ cm^−1^.

### CD spectroscopy

CD spectra were collected for the purified nanoarchaeal TP at 10 μM in 300 mM NaF, 20 mM sodium phosphate buffer, pH 8, 1 mM dithiothreitol on an Aviv 202 CD spectrometer using a 1-mm pathlength cuvette. Spectra were recorded from 195 to 260 nm at 25 °C, with 1 nm wavelength steps and the measurement at each wavelength being averaged for 30 s. A temperature melt study was carried out by increasing the temperature of the sample from 4 to 90 °C in 1 °C increments, with the ellipticity being monitored at 216 nm. The sample was then incubated at 90 °C for 2 min and cooled from 90 to 4 °C in 1 °C decrements, with the ellipticity being monitored at 216 nm.

### Tetranucleotide composition analysis

Tetranucleotide composition analysis can be used to identify core genome signatures to aid in taxonomic assignment, or to differentiate conserved protein-coding regions from those that were horizontally acquired^44^,^45^,^46^. Tetranucleotide distributions of Nanoarchaeota genomes were determined as previously described^43^, using a custom Python script. Briefly, sequences were fragmented with a 5-kb sliding window (500-bp overlapping step). Tetranucleotide frequencies were calculated by a zero-order Markov method, which applies odds ratios of observed counts for the 256 unique 4-mers, normalized to their respective mononucleotide frequencies. In order to assess tetranucleotide signatures for DGR regions (~2 kb each), while avoiding a compositional bias of flanking sequence, we concatenated DGR cassettes from both OTU1 and OTU2 and fragmented this DGR-specific sequence (~21 kb) with a sliding window as above. In addition, sequences from RT genes and TP genes were separately concatenated and fragmented with a sliding window as above to compare tetranucleotide compositions for the two DGR components. Dimensionality reduction was performed via non-metric multidimensional scaling on Euclidean distances, using the vegan package in R^47^, and ordination ellipses representing the 95% confidence region were drawn with the ‘ordiellipse()’ function.

## Additional information

**Accession codes**: Metagenomic sequence reads have been deposited in the NCBI BioSample database with accession code PRJNA47435.DV-ANM1. The ANMV-1 assembled genome sequence has been deposited in the NCBI nucleotide database with the accession code KP703175.

**How to cite this article:** Paul, B. G. *et al*. Targeted diversity generation by intraterrestrial archaea and archaeal viruses. *Nat. Commun.* 6:6585 doi: 10.1038/ncomms7585 (2015).

## Supplementary Material

Supplementary Information Supplementary Figures 1-7 and Supplementary Table 1
