# Supplementary material for: Targeted diversity generation by intraterrestrial archaea and archaeal viruses
Source: Nat Commun. 2015 Mar 23;6:6585. doi: 10.1038/ncomms7585 (PMC4372165; doi:10.1038/ncomms7585)
Supplement: Supplementary Information — Supplementary Figures 1-7 and Supplementary Table 1 [file ncomms7585-s1.pdf]

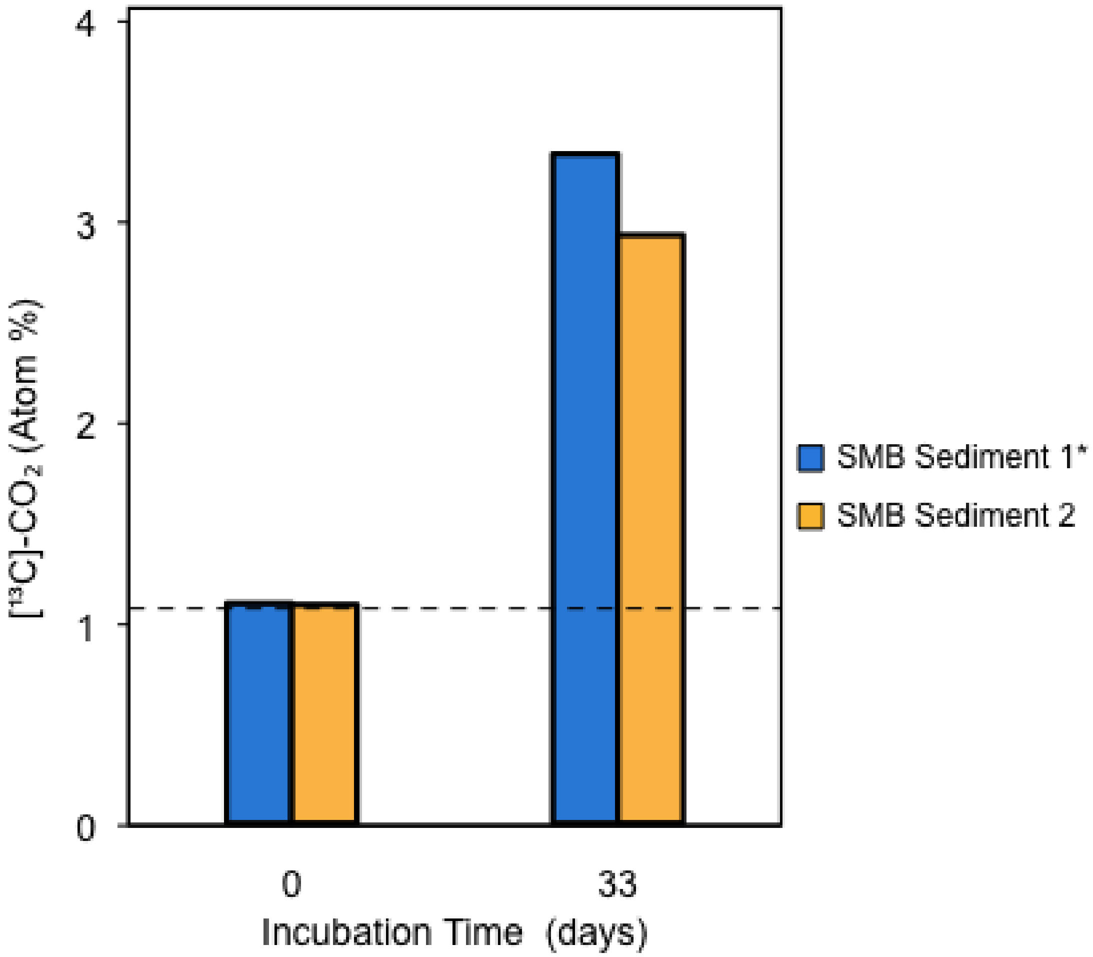

Supplementary Figure 1: Carbon isotope ratio for CO_2 in anaerobic incubations amended with ^{13}C-enriched methane. An asterisk indicates the incubation that was used for metagenomic sequencing of viral DNA in this study. The dashed line indicates natural ^{13}C abundance for CO_2 in seawater.

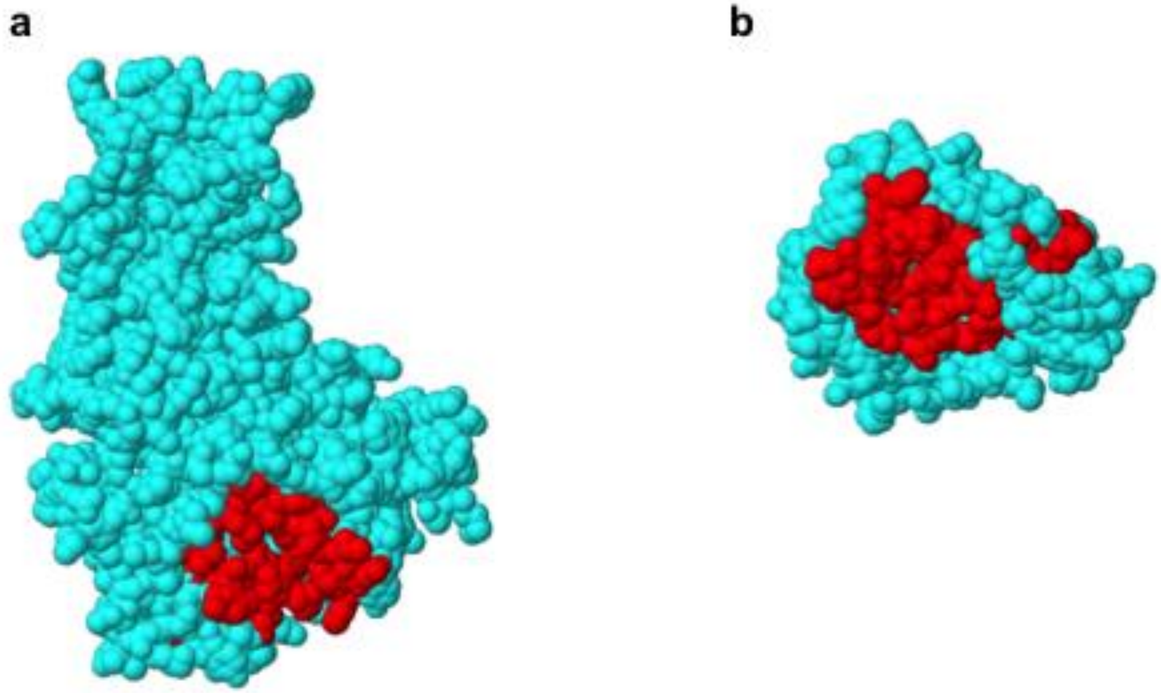

Supplementary Figure 2: **Predicted structure of ANMV-1 DGR target protein, AdtA.** a, Spacefilling diagram of AdtA (side view), modeled from *Bordetella* phage BPP Mtd. Residues that correspond to VR are shown in red. b, Spacefilling diagram of AdtA (bottom view) showing only residues aligned to Mtd with strong confidence (Phyre >90%). Exposed VR residues are highlighted in red.

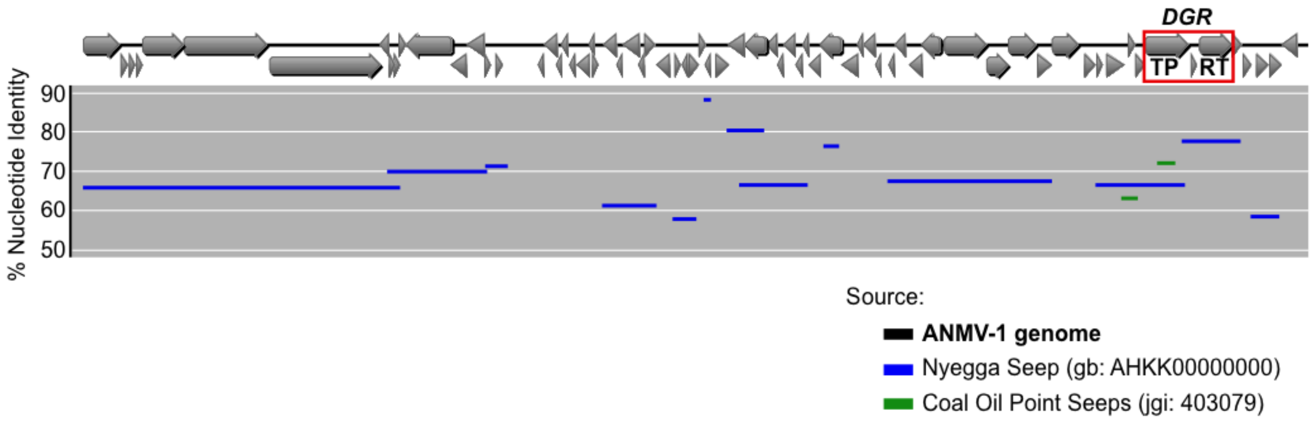

Supplementary Figure 3: Alignment of ANMV-like environmental metagenome sequences. Assembled sequences from Nyegga Seeps, offshore Norway, and Coal Oil Point, offshore California are recruited by global alignment to the ANMV-1 genome (grey arrows denote predicted ANMV ORFs). A red box indicates the DGR region of ANMV-1. Accession codes are provided for the following databases: gb, GenBank; jgi, Joint Genome Institute.

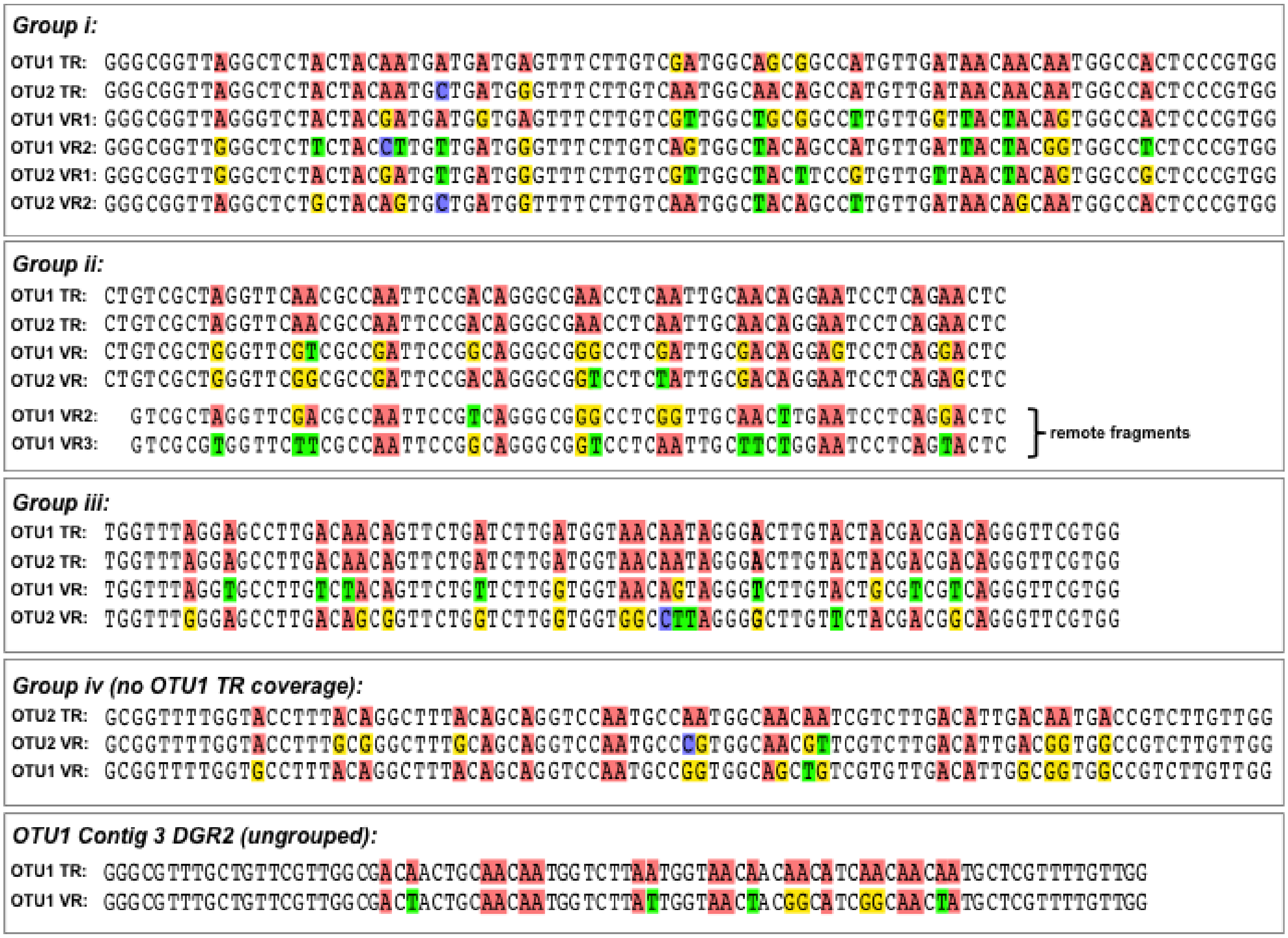

Supplementary Figure 4: Sequence alignments of immutable template (TR) and variable (VR) regions in *Nanoarchaeota* DGRs. Sequences for each homology-based DGR group are shown (see Figure 2). Adenine sites for TR are highlighted with corresponding VR sites in the nucleotide sequence.

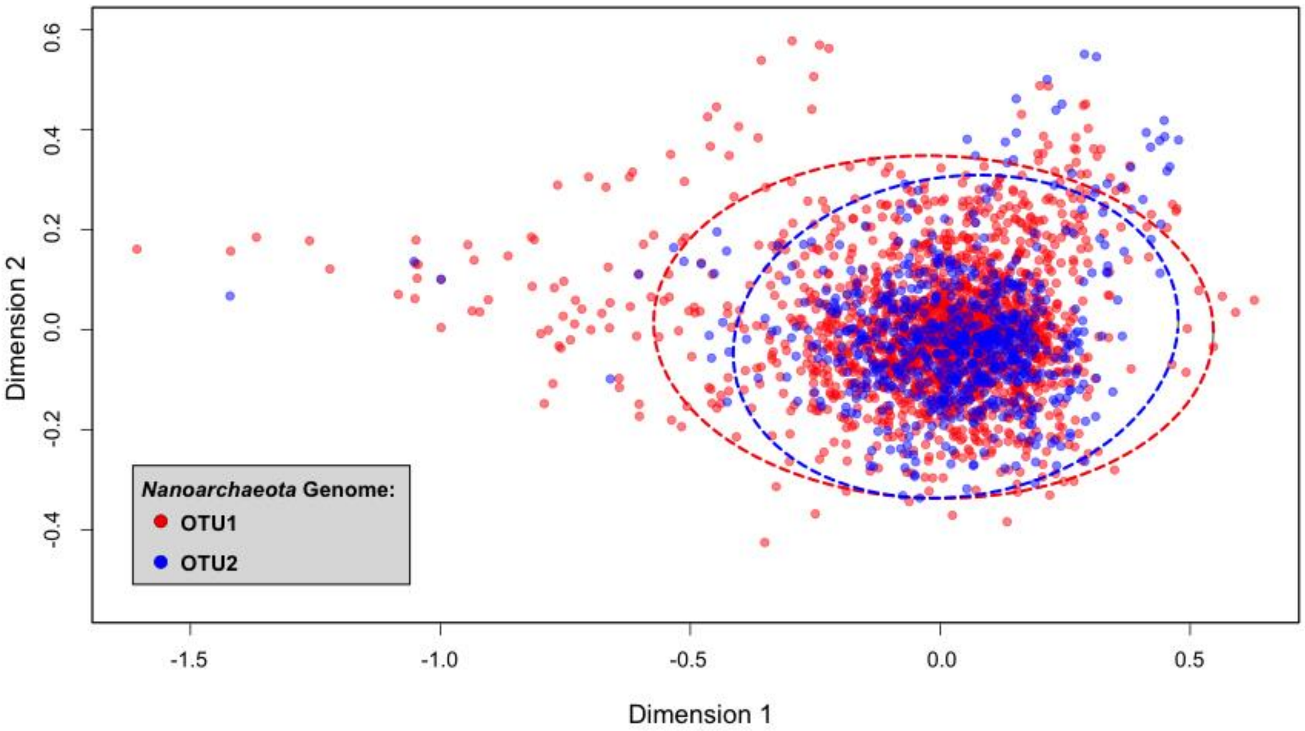

Supplementary Figure 5: Tetranucleotide signatures compared between DUSEL4 *Nanoarchaeota* OTU1 and OTU2. Colored circles represent 5kb fragments of genomic sequences. Ellipses designate 95% confidence regions corresponding to each OTU.

a

Reverse Transcriptase (% amino acid similarity)

Group		OTU1 Contig 33	OTU2 Contig 4	OTU1 Contig 27	OTU2 Contig 26	OTU1 Contig 3 (DGR1)	OTU2 Contig 13	OTU2 Contig 14	OTU1 Contig 3 (DGR2)
(i)	OTU1 Contig 33 (TP1)		98.0%	54.3%	52.5%	57.2%	56.9%	58.3%	63.4%
(i)	OTU2 Contig 4	98.0%		54.9%	53.1%	57.5%	57.2%	58.1%	63.4%
(ii)	OTU1 Contig 27	54.3%	54.9%		96.2%	53.7%	53.4%	50.9%	53.6%
(ii)	OTU2 Contig 26	52.5%	53.1%	96.2%		51.8%	51.5%	49.4%	52.0%
(iii)	OTU1 Contig 3 DGR1	57.2%	57.5%	53.7%	51.8%		99.7%	53.1%	57.4%
(iii)	OTU2 Contig 13	56.9%	57.2%	53.4%	51.5%	99.7%		53.1%	57.1%
(iv)	OTU1 Contig 26	(no RT coverage)							
(iv)	OTU2 Contig 14	58.3%	58.1%	50.9%	49.4%	53.1%	53.1%		55.5%
	OTU1 Contig 3 DGR2	63.4%	63.4%	53.6%	52.0%	57.4%	57.1%	55.5%	

b

Target Protein (% amino acid similarity)

Group		OTU1 Contig 33 (TP1)	OTU1 Contig 33 (TP2)	OTU2 Contig 4	OTU1 Contig 27	OTU2 Contig 26	OTU1 Contig 3 (DGR1)	OTU2 Contig 13	OTU1 Contig 26	OTU2 Contig 14	OTU1 Contig 3 (DGR2)
(i)	OTU1 Contig 33 (TP1)		10.2%	97.3%	5.9%	5.6%	10.5%	10.2%	8.4%	8.4%	7.9%
(i)	OTU1 Contig 33 (TP2)	10.2%		10.2%	5.1%	8.3%	8.7%	8.7%	7.2%	7.9%	8.1%
(i)	OTU2 Contig 4	97.3%	10.2%		5.9%	5.6%	10.5%	10.2%	8.4%	8.4%	7.9%
(ii)	OTU1 Contig 27	5.9%	5.1%	5.9%		34.1%	6.5%	6.5%	7.3%	7.3%	7.8%
(ii)	OTU2 Contig 26	5.6%	8.3%	5.6%	34.1%		8.5%	8.8%	8.4%	8.4%	10.6%
(iii)	OTU1 Contig 3 DGR1	10.5%	8.7%	10.5%	6.5%	8.5%		94.2%	16.4%	16.0%	11.5%
(iii)	OTU2 Contig 13	10.2%	8.7%	10.2%	6.5%	8.8%	94.2%		16.8%	16.0%	11.5%
(iv)	OTU1 Contig 26	8.4%	7.2%	8.4%	7.3%	8.4%	16.4%	16.8%		95.7%	12.7%
(iv)	OTU2 Contig 14	8.4%	7.9%	8.4%	7.3%	8.4%	16.0%	16.0%	95.7%		13.1%
	OTU1 Contig 3 DGR2	7.9%	8.1%	7.9%	7.8%	10.6%	11.5%	11.5%	12.7%	13.1%	

Supplementary Figure 6: DUSEL4 *Nanoarchaeota* DGR target and RT protein sequence comparisons. a, Similarity matrix for aligned DUSEL4 *Nanoarchaeota* DGR RTs. DGRs are colored based on shared DGR homology between OTUs. OTU1 Contig 33 contains two target proteins that are each diversified by a single RT; OTU1 Contig 26 does not contain an RT ORF. b, Distance matrix of pairwise alignments between DUSEL4 *Nanoarchaeota* DGR target proteins.

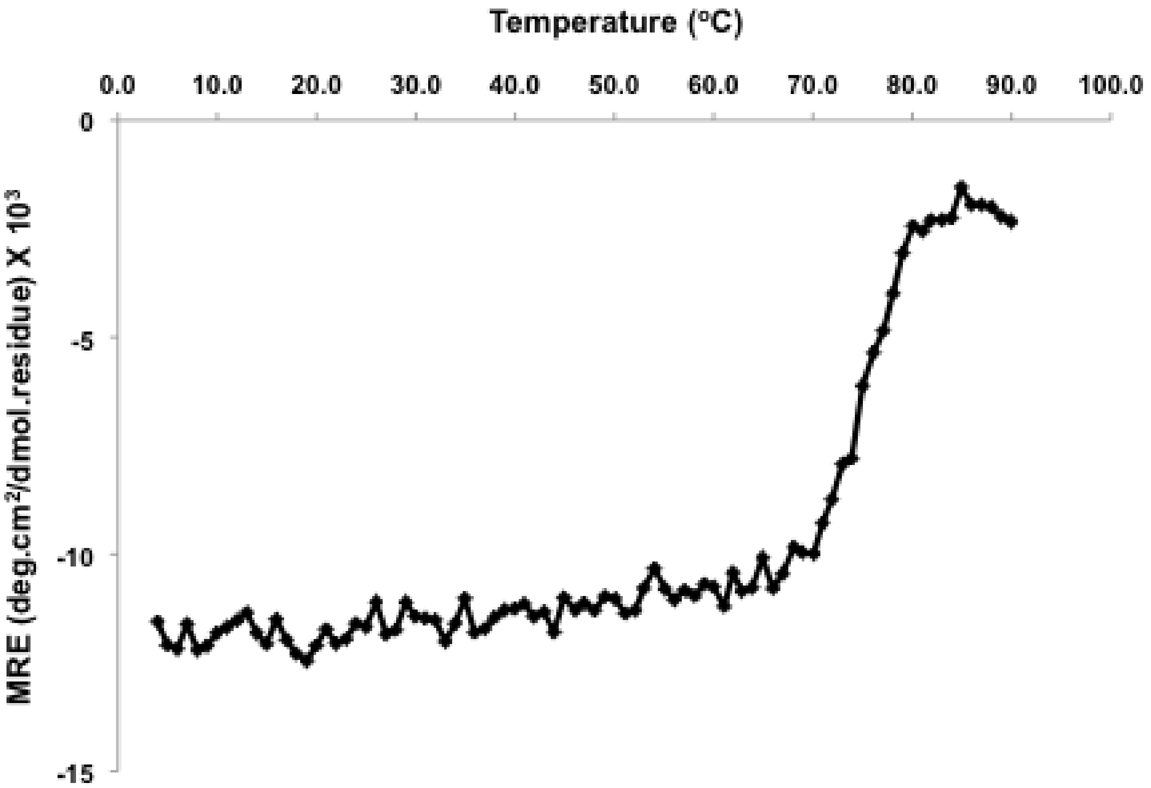

Supplementary Figure 7: CD spectra of a DGR target protein from DUSEL4 *Nanoarchaeota* (OTU1 Contig 3 DGR 2). Shown with respect to change in temperature from 4 °C to 90 °C. Mean residue ellipticity (MRE) values were measured at 216nm.

Supplementary Table 1. Gene annotations of ANMV-1. Highlighting ANME-like (orange) and DGR proteins (blue). N/A (not applicable) indicates that hits were not retrieved below an e-value threshold of 1x10-3.

ID	Start	Stop	Strand	Product:	Best-Blast Hit	e-value	Best ANME Hit	ANME Hit e-value	Database
ANMV-1_1	22	1,176	+	hypothetical protein	N/A	N/A	N/A	N/A	N/A
ANMV-1_2	1,224	1,448	+	hypothetical protein	N/A	N/A	N/A	N/A	N/A
ANMV-1_3	1,476	1,664	+	hypothetical protein	N/A	N/A	N/A	N/A	N/A
ANMV-1_4	1,661	1,885	+	hypothetical protein	N/A	N/A	N/A	N/A	N/A
ANMV-1_5	1,913	3,193	+	MutT-like phage hydrolase	Enterococcus faecalis V583	2.00E-11	N/A	N/A	ACLAME
ANMV-1_6	3,200	5,800	+	ATP-dependent DNA ligase	Cellulomonas flavigena DSM 20109	9.00E-06	N/A	N/A	NCBI nr
ANMV-1_7	5,868	9,368	+	hypothetical protein	N/A	N/A	N/A	N/A	N/A
ANMV-1_8	9,356	9,640	-	hypothetical protein	N/A	N/A	N/A	N/A	N/A
ANMV-1_9	9,702	9,830	+	hypothetical protein	N/A	N/A	N/A	N/A	N/A
ANMV-1_10	9,835	9,984	+	hypothetical protein	N/A	N/A	N/A	N/A	N/A
ANMV-1_11	9,953	10,162	+	conserved hypothetical protein (RNAse-H domain)	Methanolobus psychrophilus R15	5.60E-16	uncultured archaeon ANME-1	8.40E-16	NCBI nr
ANMV-1_12	10,185	11,606	-	Phage terminase large subunit	Candidatus Methanoperedens nitroreducens ANME-2D	6.30E-26	(best blast hit)	(best blast hit)	NCBI nr
ANMV-1_13	11,596	12,057	-	hypothetical protein	N/A	N/A	N/A	N/A	N/A
ANMV-1_14	12,057	12,650	-	TATA-box binding protein	Vulcanisaeta distributa DSM 14429	1.70E-08	uncultured archaeon ANME-1	1.90E-07	NCBI nr
ANMV-1_15	12,973	13,200	+	hypothetical protein	N/A	N/A	N/A	N/A	N/A
ANMV-1_16	14,349	14,471	-	hypothetical protein	N/A	N/A	N/A	N/A	N/A
ANMV-1_17	14,497	14,922	-	hypothetical protein	Candidatus Methanoperedens nitroreducens ANME-2D	5.40E-13	(best blast hit)	(best blast hit)	NCBI nr
ANMV-1_18	14,919	15,038	-	hypothetical protein	N/A	N/A	N/A	N/A	N/A
ANMV-1_19	15,044	15,256	-	hypoethical protein	Gloeophyllum trabeum ATCC 11539	1.60E-05	N/A	N/A	NCBI nr
ANMV-1_20	15,305	15,511	-	hypothetical protein	N/A	N/A	N/A	N/A	N/A
ANMV-1_21	15,517	15,939	-	hypothetical protein	N/A	N/A	N/A	N/A	N/A
ANMV-1_22	15,936	16,049	-	hypothetical protein	N/A	N/A	N/A	N/A	N/A
ANMV-1_23	16,053	16,169	+	hypothetical protein	N/A	N/A	N/A	N/A	N/A
ANMV-1_24	16,371	16,799	-	hypothetical protein	N/A	N/A	N/A	N/A	N/A
ANMV-1_25	16,956	17,498	-	hypothetical protein	N/A	N/A	N/A	N/A	N/A
ANMV-1_26	17,488	17,661	-	hypothetical protein	N/A	N/A	N/A	N/A	N/A
ANMV-1_27	17,654	17,980	+	hypothetical protein	N/A	N/A	N/A	N/A	N/A
ANMV-1_28	18,042	18,437	-	hypothetical protein	N/A	N/A	N/A	N/A	N/A
ANMV-1_29	18,655	18,792	+	hypothetical protein	N/A	N/A	N/A	N/A	N/A
ANMV-1_30	18,860	18,991	-	hypothetical protein	N/A	N/A	N/A	N/A	N/A
ANMV-1_31	19,055	19,354	+	hypothetical protein	N/A	N/A	N/A	N/A	N/A
ANMV-1_32	19,354	19,590	+	hypothetical protein	N/A	N/A	N/A	N/A	N/A
ANMV-1_33	19,557	19,754	-	hypothetical protein	N/A	N/A	N/A	N/A	N/A
ANMV-1_34	19,903	20,322	+	hypothetical protein	N/A	N/A	N/A	N/A	N/A
ANMV-1_35	20,293	20,826	-	hypothetical protein	uncultured marine thaumarchaeote AD1000	1.30E-23	N/A	N/A	NCBI nr
ANMV-1_36	20,823	21,485	-	Phage gp37/gp68	Mahella australiensis 50-1 BON	2.00E-30	N/A	N/A	NCBI nr
ANMV-1_37	21,482	21,655	-	hypothetical protein	N/A	N/A	N/A	N/A	N/A
ANMV-1_38	21,847	22,026	-	hypothetical protein	N/A	N/A	N/A	N/A	N/A
ANMV-1_39	22,019	22,414	-	hypothetical protein	N/A	N/A	N/A	N/A	N/A
ANMV-1_40	22,414	22,629	-	hypothetical protein	N/A	N/A	N/A	N/A	N/A
ANMV-1_41	22,622	22,819	-	hypothetical protein	N/A	N/A	N/A	N/A	N/A
ANMV-1_42	22,816	23,190	-	hypothetical protein	N/A	N/A	N/A	N/A	N/A
ANMV-1_43	23,174	23,845	-	Crossover junction endodeoxyribonuclease	Salmonella enterica	2.50E-06	N/A	N/A	ACLAME
ANMV-1_44	23,838	24,377	-	hypothetical protein	N/A	N/A	N/A	N/A	N/A
ANMV-1_45	24,374	24,532	-	hypothetical protein	N/A	N/A	N/A	N/A	N/A
ANMV-1_46	24,537	24,926	-	hypothetical protein	N/A	N/A	N/A	N/A	N/A
ANMV-1_47	24,923	25,084	-	hypothetical protein	N/A	N/A	N/A	N/A	N/A
ANMV-1_48	25,340	25,519	-	hypothetical protein	N/A	N/A	N/A	N/A	N/A
ANMV-1_49	25,525	25,959	-	Anaerobic ribonucleoside triphosphate reductase	uncultured bacterium	6.00E-15	N/A	N/A	NCBI nr
ANMV-1_50	25,963	26,376	-	hypothetical protein	uncultured archaeon GZfos37D1 (ANME1)	6.05E-07	(best blast hit)	(best blast hit)	NCBI nr
ANMV-1_51	26,351	26,959	-	hypothetical protein	uncultured archaeon GZfos37D1 (ANME1)	4.80E-13	(best blast hit)	(best blast hit)	NCBI nr
ANMV-1_52	27,069	28,388	+	prohead protease	Alkaliphilus metalliredigens QYMF	5.00E-08	N/A	N/A	ACLAME
ANMV-1_53	28,388	29,074	+	hypothetical protein	N/A	N/A	N/A	N/A	N/A
ANMV-1_54	29,071	29,964	+	hypothetical protein	N/A	N/A	N/A	N/A	N/A
ANMV-1_55	29,983	30,438	+	hypothetical protein	N/A	N/A	N/A	N/A	N/A
ANMV-1_56	30,456	31,277	+	hypothetical protein	N/A	N/A	N/A	N/A	N/A
ANMV-1_57	31,471	31,821	+	hypothetical protein	N/A	N/A	N/A	N/A	N/A
ANMV-1_58	31,874	32,035	+	hypothetical protein	N/A	N/A	N/A	N/A	N/A
ANMV-1_59	32,161	32,742	+	Capsid tail fiber	Desulfotomaculum reducens MI-1	4.00E-07	N/A	N/A	ACLAME
ANMV-1_60	32,831	33,058	+	Capsid tail fiber	Desulfotomaculum reducens MI-1	2.20E-07	N/A	N/A	ACLAME
ANMV-1_61	33,107	33,388	+	hypothetical protein	N/A	N/A	N/A	N/A	N/A
**ANMV-1_62**	**33,388**	**34,728**	**+**	**DGR target protein adtA**	**N/A**	**N/A**	**N/A**	**N/A**	**N/A**
**ANMV-1_63**	**34,834**	**35,028**	**+**	**hypothetical protein (putative Avd)**	**N/A**	**N/A**	**N/A**	**N/A**	**N/A**
**ANMV-1_64**	**35,045**	**36,103**	**+**	**Reverse transcriptase**	**Nanoarchaeota archaeon SCGC AAA011-L22**	**1.70E-82**	**N/A**	**N/A**	**NCBI nr**
ANMV-1_65	36,106	36,402	+	hypothetical protein	N/A	N/A	N/A	N/A	N/A
ANMV-1_66	36,445	36,714	+	hypothetical protein	N/A	N/A	N/A	N/A	N/A
ANMV-1_67	36,857	37,243	+	hypothetical protein	N/A	N/A	N/A	N/A	N/A
ANMV-1_68	37,306	37,668	+	hypothetical protein	N/A	N/A	N/A	N/A	N/A
ANMV-1_69	37,670	38,170	-	hypothetical protein	N/A	N/A	N/A	N/A	N/A
